# Influence of Plant Growth Regulators and Artificial Light on the Growth and Accumulation of Inulin of Dedifferentiated Chicory (*Cichorium intybus* L.) Callus Cells

**DOI:** 10.3390/life12101524

**Published:** 2022-09-29

**Authors:** Rima N. Kirakosyan, Anton V. Sumin, Anna A. Polupanova, Maria G. Pankova, Irina S. Degtyareva, Nikolay N. Sleptsov, Quyet V. Khuat

**Affiliations:** 1 Department of Biotechnology, Russian State Agrarian University—Moscow Timiryazev Agricultural Academy, Timiryazevskaya Str., 49, 127550 Moscow, Russia; 2 Department of Plant Physiology, Russian State Agrarian University—Moscow Timiryazev Agricultural Academy, Timiryazevskaya Str., 49, 127550 Moscow, Russia; 3 Biology and Agricultural Engineering Faculty, Hanoi Pedagogical University 2, Nguyen Van Linh, Phuc Yen 15000, Vietnam

**Keywords:** artificial light, auxins, chicory, callus cells, inulin, plant growth regulators

## Abstract

Chicory (*Chicorium intybus* L.) is a perennial herb of the family *Asteraceae*, widely distributed in Asia and Europe, commonly used industrially as a raw material for extracting inulin because of a high content of inulin and biologically active compounds. Light conditions and plant growth regulators (PGRs) are two of many factors that affect the growth and inulin content of chicory callus. The aim of this work is to study the effect of PGRs and light conditions on proliferation and accumulation of inulin of chicory callus in vitro. In this study, we used semi-solid MS medium supplemented with different auxins (including Indole-3-acetic acid (IAA), naphthylacetic acid (NAA), and 2,4-dichlorophenoxyacetic acid (2,4-D)) at a concentration of 5.5–9.5 mg/L in combination with 2.0 mg/L 6 benzylaminopurine (BA) to determine induction and proliferation of callus. The increasing value of callus fresh weight was used to assess the growth of the callus in treatments. The results showed that a steady increase in callus fresh weight and inulin content in callus cells was obtained when they were cultured on MS medium supplemented with a combination of 2.0 mg/L BA with 7.5 mg/L IAA in lighting conditions with radiation equalized by the flux density of photosynthetic photons and ratios of radiation levels in the region of FR—far red > R—red. Increasing demand for organic inulin sources in production practice can be met by our finding.

## 1. Introduction

The production of high-quality drugs, characterized by safety and high efficiency, is one of the priority areas for the development of the pharmaceutical industry. The production of such drugs is based on the use of plant materials, in particular medicinal plants. It plays an important role in expanding the range of medicinal products [1,2,3]. According to the World Health Organization (WHO) forecast and the WHO Traditional Medicine Strategy 2014–2023, in 15–20 years, the share of herbal medicines in the total range of medicines may increase to 60% [4].

The interest of researchers in medicinal plants is constantly growing, since they are a source of biologically active substances that can be widely used in the food industry as well. Today, special attention is paid to the production of food for dietary and functional purposes, which include, for example, dietary fiber, antioxidants, prebiotics, etc. [5]. One of the effective prebiotics is inulin, which is industrially most often extracted from chicory (*Cichorium intybus* L.) [6]. Inulin is a heterogeneous collection of fructose polymers. It consists of chain-terminating glucosyl moieties and a repetitive fructosyl moiety, which are linked by β(2,1) bonds [7]. The degree of polymerization (DP) of standard inulin ranges from 2 to 60 [8,9]. Because of the β(2,1) linkages, inulin is not digested by enzymes in the human alimentary system, contributing to its functional properties: reduced calorie value, dietary fiber, and prebiotic effects [10].

Chicory is a perennial herb of the family *Asteraceae*, widely distributed in Asia and Europe [11,12], commonly used industrially as a raw material for extracting inulin because of a high content of inulin and biologically active compounds. All parts of this plant possess great medicinal importance due to the presence of a number of medicinally important compounds such as alkaloids, inulin, sesquiterpene lactones, coumarins, vitamins, chlorophyll pigments, unsaturated sterols, flavonoids, saponins and tannins [13,14,15,16]. According to Meehye and Shin (1996) [17], fresh chicory typically contains 68% inulin, 14% sucrose, 5% cellulose, 6% protein, 4% ash, and 3% other compounds, while dried chicory contains approximately 98% inulin and 2% other compounds. Chicory has been traditionally used for the treatment of fever, diarrhea, jaundice, and gallstones [18,19]. Several recent studies have been also reported that chicory has a potent hepatoprotective, antioxidant, hypoglycemic, diuretic, anti-testicular toxicity, and immunomodulatory effects [20,21,22,23,24]. Moreover, its roots are often used as a coffee substitute [25], particularly in India [26] and South Africa.

Nowadays, the use of biotechnology methods not only allows to multiply and obtain high-quality planting material but also creates in vitro cell cultures of medicinal plants with an increased content of biologically active substances [27,28]. Several previous reports have demonstrated the regenerative ability of chicory from different plant parts (including leaf explant [29,30,31,32,33,34,35], and petiole explant [36,37] by using different hormonal combinations. In most of the reported studies, leaf explants were used most in micropropagation through callus cultures, and combinations of BA with IAA or NAA at different concentrations were used for the initial induction and proliferation of the callus [31,32,33,35]. In addition, light is known to be an effective abiotic elicitor that influences plant photosynthesis process, development, and morphogenesis [38,39,40]. Light plays a vital role in regulating primary as well as secondary metabolism to help achieve optimum growth [41,42]. Multiple studies have reported the direct stimulation of secondary metabolites production in the presence of monochromatic lights, especially red light [43,44,45]. There are currently no similar reports on chicory species.

Based on the foregoing, the aim of this work is to study the effect of plant growth regulators (PGRs) and light conditions on proliferation and accumulation of inulin of Chicory (*Cichorium intybus* L.) callus in vitro.

## 2. Materials and Methods

### 2.1. Plant Material Prepareation

The work was carried out at the Department of Biotechnology of the Russian State Agrarian University—Moscow Agricultural Academy named after K. A. Timiryazev (Moscow, Russia). The objects of the study were leaf segments obtained from in vitro seedlings of Chicory (*Cichorium intybus* L.), cultivar Petrovsky.

Seeds were sterilized with a 0.1% mercuric chloride (HgCl_2_) solution for 9 min, followed by rinsing them three times with sterile distilled water following the protocol described in the literature data [35].

All work on sterilization of seeds, introduction into culture in vitro and further work on the study of callogenesis and morphogenesis were carried out in aseptic conditions of laminar hood flow (BIOBASE BBS—H1800(X)). After surface disinfection, seeds were cultivated on a PGR-free nutrient medium of Murashige and Skoog (MS) [46]. The pH of the medium was adjusted to 5.6–5.8 before being autoclaved at 121 °C and 1.1 atm for 20 min. The cultures were maintained in a culture room at 25 ± 2 °C during a long-day photoperiod (16 h of light: 8 h of dark) with cool white fluorescent light (2000–2500 lux). Five seeds were cultured per Petri dish and ten dishes per treatment.

Seedlings obtained on the 7th day from the time the seeds germinated were subcultured to MS medium supplemented with 1.0 mg/L 6–benzylaminopurine (BA) and 0.1 mg/L Indole–3–acetic acid (IAA) for in vitro plantlet development.

### 2.2. Cultivation of Chicory Callus under Different PGR Composition

In order to induce callus, leaf segments (5 × 5 mm) isolated from 30 days old in vitro plantlets were cultivated in MS medium supplemented with cytokinin (2.0 mg/L BA) and various auxins (including IAA, naphthylacetic acid (NAA), and 2,4-dichlorophenoxyacetic acid (2,4-D)) at a concentration of 5.5–9.5 mg/L. Ten leaf explants were cultured per Petri dish and fifteen dishes per treatment.

Every 4 weeks, the callus was transferred onto a fresh medium. Five calluses were cultured per Petri dish and thirty dishes per treatment.

The consistency and color of the callus were taken into account. The increasing value of callus fresh weight was used to assess the growth of the callus. Biomass of callus was weighed on scales (AND GR-202) in a laminar flow hood.

### 2.3. Cultivation of Chicory Callus under Light-Culture Conditions

Chicory leaf segments obtained from 30 days old in vitro plantlets were cultivated on semisolid MS medium supplemented with 2.0 mg/L BA in combination with 7.5 mg /L NAA or 7.5 mg /L IAA. Ten leaf explants were cultured per Petri dish and fifteen dishes per treatment. Petri dishes were placed in light proof grow tents (Urban Grower 60 × 60 × 200 cm (Gorshkoff, Russia)) with radiation equalized by the flux density of photosynthetic photons and different ratios of radiation levels in the region of 660 nm (R—red) and 730 nm (FR—far red).

R/FR ratio options:

(1) R/FR = 1, photosynthetic photon flux density (PPFD) = 142 (±10) μmol/m^2^s;
(2) R/FR = 1/2, PPFD = 142 (±10) μmol/m^2^s;
(3) R/FR = 2, PPFD = 142 (±10) μmol/m^2^s;
(4) Control: white linear fluorescent lamp (OSRAM AG 4000K), PPFD 40 μmol/m^2^s.

The emission spectra were measured with a PLA-20 instrument (Everfine, Hangzhou, China).

The increasing value of callus fresh weight was used to assess the growth of the callus. The callus was weighed in a laminar flow hood by scales at the beginning and the end of the subculture.

### 2.4. Determination of Inulin by Spectrophotometry

For one analysis, a dry sample of 600 mg callus was taken, placed in a volumetric flask, poured in 9.5 mL of 90% ethanol, and kept for 30 min in a water bath (UCHEN) at 80 °C, periodically stirring its content. As a control, the same volume of water was added to the sample of callus.

After cooling, 0.05 mL of 25% NaOH solution was added to both flasks (sample and control) and then the contents of them were brought to 10 mL with ethanol at the sample flask and water at the control flask. The contents of the flasks were left for 10–20 min after which centrifugation was carried out at 4000–6000 rpm for 3–5 min. After that, empty 10 mL volumetric flasks were taken and 0.05 mL of centrifugates of both extracts (sample and control), as well as a solution of 3.0 mg/mL fructose (standard) and water (control), were transferred into them. In the next step, the resulting volume was added to 1.0 mL of reagent (2.0 mg/mL resorcinol + 96% ethanol and concentrated hydrochloric acid (HCl) in equal volumes). The flasks were kept in a water bath for 35 min and then cooled. The volume was brought to 10 mL with water and stirred.

The optical density of the analyzed samples and standard solution was measured on an SF-104 spectrophotometer at a wavelength of 480 nm. The content of fructose-containing sugars in the samples was calculated using the formula:

(1) $$X=\frac{A_{p}\times C_{s}\times 10}{A_{s}\times m_{p}}$$

where *A_p_* and *A_s_* are the optical density of the test sample and the standard solution, respectively; *C_s_* is the concentration of a standard fructose solution (3.0 mg/mL); *m_p_* is the weight of the weighed portion of the analyzed sample, g.

The result obtained for an aqueous extract reflects the total content of water-soluble carbohydrates. The result for the ethanol extract reflects the content of low molecular weight fructosides. The difference between the two measures gives the inulin content. [47,48].

### 2.5. Statistical Analysis of Experimental Data

The experiments were arranged completely randomly and repeated three times. Mean values of all data were calculated using Microsoft Excel 2013 (Microsoft Corporation, Redmond, WA, USA). Analysis of Variance (ANOVA) was performed in AGROS software (version 2.11, Russian State Agrarian University—Moscow Timiryazev Agricultural Academy, Moscow, Russia, 1999–2001) and means were compared using Fisher’s Least Significant Difference (LSD) test at a significance level of *p* ≤ 0.05.

## 3. Results

### 3.1. Influence of the PGR Composition of the Nutrient Medium on Chicory Callus Growth

Some patterns have been established in chicory callus formation, as a result of the studies carried out. On days 7 to 10, the proliferation of cells was observed at the sites of cut and damage in all treatments. However, it should be noted that the applied different concentrations of various auxins (IAA, NAA, and 2,4-D) in combination with 2.0 mg/L BA had a significant effect on the growth, texture, and color of callus.

**Figure 1:**
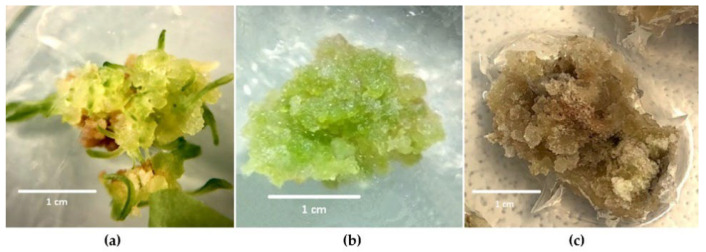
Formation callus on semisolid MS medium supplemented with different concentrations of various auxins (IAA, NAA, and 2,4-D) in combination with 2.0 mg/L BA: (**a**) 7.5 mg/L IAA; (**b**) 7.5 mg/L NAA; (**c**) 5.5 mg/L 2,4-D. Callus after 28 days of culture (the first subculture). Scale bars = 1 cm.

Cultivation of leaf explants on semisolid MS medium supplemented with different concentrations of IAA in combination with 2.0 mg/L BA led to the callus formation of a bright yellow color, semisolid, and with the formation of meristematic foci (Figure 1a). The onset of callogenesis was noted on the third day from the beginning of culture. With NAA, the chicory callus was dense and white or light yellow (Figure 1b). The onset of callogenesis was observed on days 7–10. When leaf explants were cultivated on a medium containing 2,4-D, a different result was observed. In this variant, the intensity of callogenesis was minimal. The formed callus was brown, of loose consistency, and died during cultivation. Therefore, the culture medium containing 2,4-D was not used in further experiments (Figure 1c).

The increasing value of callus fresh weight was used to assess the growth of the callus. This indicator was evaluated at fourth and fifth subcultures. The growth of callus was determined depending on the investigated auxin and its concentration (Table 1).

The stable growth of callus was observed when NAA was added to the nutrient medium at a concentration of 7.5 and 8.5 mg/L at the fifth subculture (reach 1.24 g) and 6.5 mg/L at the fourth subculture (reach 1.56 g). In the treatment with IAA (at the concentration of 8.5 mg/L in combination with 2.0 mg/L BA), the increasing value of callus fresh weight is about two times less than in the treatment with NAA at the same concentration. However, it should be noted that, on a medium containing NAA, the proliferative activity decreased with an increase in the number of subcultures. A stable increase in callus cells was observed between fourth and fifth subculture during all the IAA treatments. In addition, in these treatments in the callus at the end of the cultivation cycle, the formation of multiple meristematic foci was noted.

The difference in the growth of callus during subculturing was revealed. When using IAA, the greatest difference in growth between the fourth and fifth subcultures was at a concentration of 7.5 mg/L and amounted to 65%. At the same time, the greatest difference in growth between NAA transfers was at the level of 7.5 mg/L and amounted to 12.9%. Combined with fresh weight, this was the best result for this PGR. Therefore, it was decided to use these PGR concentrations for further experiments.

### 3.2. Influence of Light Quality and PGR Composition on the Morphology of the Chicory Callus

One of the regulatory factors of morphogenesis is the intensity and quality of light. The paper studied the influence of ratios of red LED lamps (R = 660 nm) and far-red spectrum (FR = 730 nm) on the formation of callus from leaf segments of chicory seedlings. Various auxins (IAA or NAA) at the concentration of 7.5 mg/L in combination with 2.0 mg/L BA were used in all variants of nutrient media. It was found that under different light growth conditions, callus of different size, density, and color was formed.

**Figure 2:**
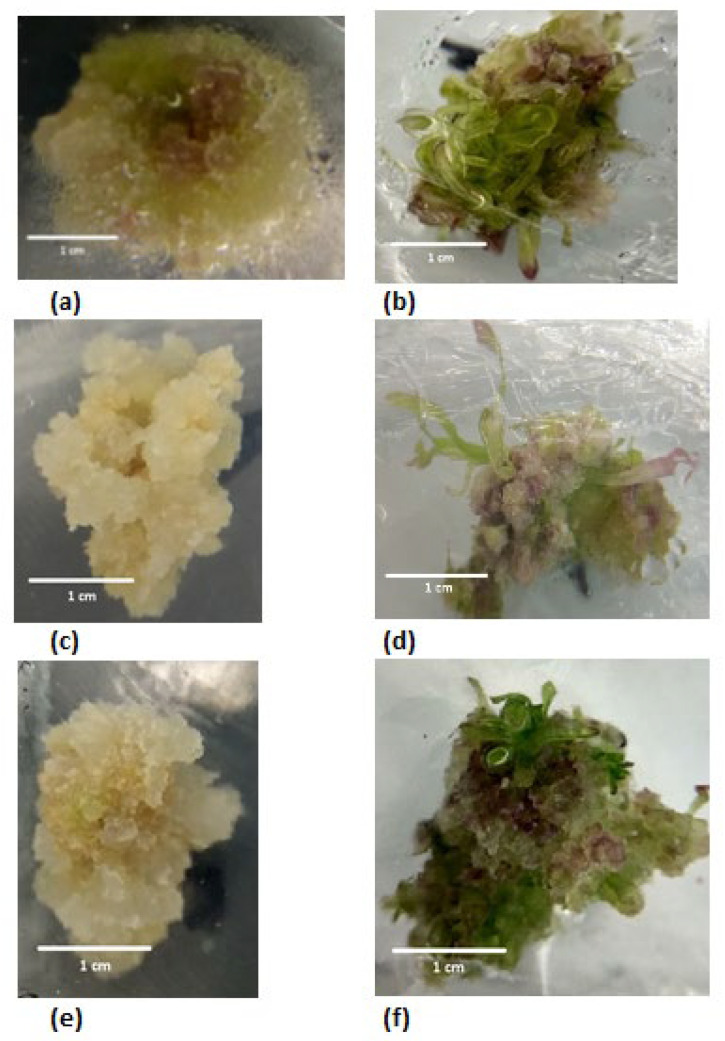
Callus obtained by cultivation on semisolid MS medium supplemented with 2.0 mg/L BA in combination with: (**a**) FR > R mode, 7.5 mg/L NAA; (**b**) FR > R mode, 7.5 mg/L IAA; (**c**) FR = R mode, 7.5 mg/L NAA; (**d**) FR = R mode, 7.5 mg/L IAA; (**e**) FR < R mode, 7.5 mg/L NAA; (**f**) FR < R mode, 7.5 mg/L IAA. Callus after 28 days of culture (the first subculture). Scale bars = 1 cm.

Growing callus under the conditions of a light regime FR > R on semisolid MS medium supplemented with 7.5 mg/L NAA in combination with 2.0 mg/L BA resulted in the formation of a loose, highly hydrated callus, and easily disintegrating into individual cell aggregates. In this case, the obtained callus was yellow and there was an anthocyanin coloration that appeared towards the center (Figure 2a). On semisolid MS medium supplemented with 7.5 mg /L IAA in combination with 2.0 mg/L BA, the obtained callus was green in color, of a dense type, and formed many meristematic foci over the entire surface of the callus (Figure 2b).

When callus was grown under the conditions of the FR = R light regime on semisolid MS medium supplemented with 7.5 mg /L NAA in combination with 2.0 mg/L BA, a loose callus type, poorly hydrated, and easily disintegrating into individual cell aggregates, was formed. In this case, the obtained callus was light yellow or white without anthocyanin patches (Figure 2c). On semisolid MS medium supplemented with 7.5 mg /L IAA in combination with 2.0 mg/L BA, the obtained callus was light green color with an anthocyanin coloration, of a dense type, and formed many meristematic foci over the entire surface of the callus (Figure 2d). However, the formed adventive shoots had a lanceolate leaf blade, which is atypical for plantlets with normal morphology.

When callus was grown under the conditions of the light regime FR < R semisolid MS medium supplemented with 7.5 mg /L NAA in combination with 2.0 mg/L BA, a loose callus type, poorly hydrated, and easily disintegrating into individual cell aggregates, was formed. In this case, the obtained callus was light yellow or white without anthocyanin patches (Figure 2e). On semisolid MS medium supplemented with 7.5 mg /L IAA in combination with 2.0 mg/L BA, callus of a bright green color with an anthocyanin coloration, of a medium density type, and formed many meristematic foci over the entire surface of the callus (Figure 2f). However, the adventive shoots formed were hyperhydrated.

### 3.3. Influence of the PGR Composition of the Nutrient Medium on the Accumulation of Inulin in Chicory Callus

The results indicated that the ability of callus cells to accumulate inulin depends on the type of auxins used. The presence of 2.0 mg/L BA in combination with 7.5 mg/L IAA in the nutrient medium led to the accumulation of inulin in the callus five times higher than 2.0 mg/L BA in combination with 7.5 mg/L NAA (Table 2). The ratio was consistent on both the fourth and fifth subcultures. The increased content of inulin in callus obtained on semisolid MS medium supplemented with 2.0 mg/L BA in combination with 7.5 mg/L IAA can be explained by the appearance of meristematic foci, in contrast to the medium with NAA, on which non-morphogenic callus was formed.

### 3.4. Influence of Light Quality and PGR Composition on the Accumulation of Inulin in Chicory Callus

**Figure 3:**
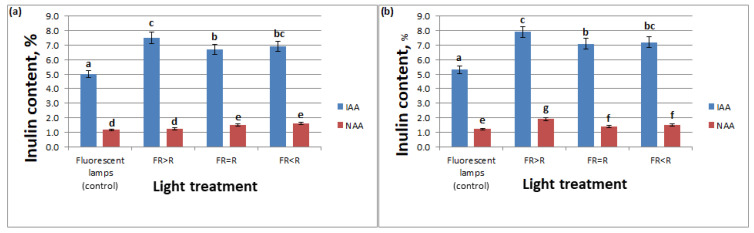
Combined effect of light quality (FR > R, FR = R, FR < R) and plant growth regulators (2.0 mg /L BA in combination with 7.5 mg/L NAA) on inulin content of chicory callus at: (**a**) Fourth subculture; (**b**) Fifth subculture. Means (percentage of inulin content) followed by a different letter are significantly different at an alpha level of 0.05 according to the Fisher’s Least Significant Difference (LSD) test. For each treatment *n* = 150.

With regard to the influence of the spectral composition of light on the accumulation of inulin in chicory callus cells, there was a clear dependence of the accumulation of inulin on the light quality. On semisolid MS medium supplemented with 7.5 mg /L IAA in combination with 2.0 mg/L BA, the responsiveness of the callus to changes in light treatment was observed. With an increase in PPFD by a mean of 100 μmol/m^2^s, all treatments (PPFD = 142 ± 10 μmol/m^2^s) differed from the control (fluorescent lamp, PPFD = 40 μmol/m^2^s) by a mean of 28%. The FR = R and FR < R treatments did not differ from each other during all subcultures. There was no difference between FR > R and FR<R as well. The effects of both the 4thand 5th subcultures were the same (Figure 3).

During the cultivation on semisolid MS medium supplemented with 7.5 mg/L NAA in combination with 2.0 mg/L BA, because the obtained callus was non-morphogenic, their weak responsiveness to lighting was observed in all treatments and control. At the fourth subculture, the R = FR and R < FR treatments were not significantly different from each other but different from the control and the FR > R treatment (Figure 3a). There was a significant difference in all treatments with control at the fifth subculture (Figure 3b). There were no differences between FR = R, FR < R. The treatment with FR > R at 5th subculture had the highest value of inulin content by a mean of 35% higher than the control (fluorescent lamp).

The high ability of callus cells to synthesize and accumulate inulin was obtained by growing cells on a nutrient medium containing IAA. Probably, the high biosynthetic potential of cells for inulin synthesis is due to the fact that it was under these conditions that a well-proliferating and highly morphogenic callus was formed.

## 4. Discussion

Currently, the most promising area for the development of the food industry is the production of functional and dietary food products. These products include products containing dietary fiber, antioxidants, prebiotics, etc. One of the effective prebiotics is inulin, which is found in chicory, which determines the importance of this crop for the food and pharmaceutical industries.

The use of biotechnology methods not only allows to multiply and obtain high-quality planting material but also creates in vitro cell cultures of medicinal plants with an increased content of biologically active substances [27,28]. The biosynthetic potential of cultured cells in vitro depends on various factors, which include, in particular: the mineral and hormonal composition of the nutrient medium as well as the use of various elicitors (physical and chemical nature) and illumination conditions (light intensity, spectral composition of light). A change in each of these factors can lead to a change in primary and secondary metabolism, and the quality of the obtained medicinal plant raw materials also depends on illumination conditions [38,39,40,41,42,43,44,45].

In most of the reported studies, leaf explants were used most in micropropagation through callus cultures, and combination of BA with IAA or NAA at different concentrations was most suitable for the initial induction and proliferation of chicory callus [31,32,33,35]. In our studies, the most optimal conditions for callogenesis were the presence of IAA or NAA in combination with 2.0 mg/L BA in the nutrient medium at a concentration of 7.5 and 8.5 mg/L. The use of 2,4-D in any of the treatments did not lead to the formation of a well-proliferating callus. In all treatments, the formation of weakly growing, non-viable tissue was observed, the death of which was observed already in the middle of the first subculture. Cultivation of callus cells on a nutrient medium supplemented with IAA or NAA led to different responses to morphogenesis. Differences in the effect of various auxins on the content of inulin, apparently, were due to the nature of the auxin. IAA is a natural auxin and NAA is a synthetic. IAA is preferred for the formation of morphogenic callus. When using NAA, the formation of a non-morphogenic callus was observed. In our study on medium with IAA, where the morphogenic callus formed, a greater amount of inulin was observed (Table 2). According to other researchers, the content of inulin in callus obtained on a medium with NAA is relatively low. However, the content of inulin in plant leaves in vitro can reach up to 5% dry weight [49]. In our case, it can be assumed that the high content of inulin in the callus on the medium with IAA is due to the formation of meristematic foci over the entire surface of the callus. Its further increase can be controlled by changing the spectral composition and quality of light. During cultivation on a medium supplemented with NAA, the callus was non-morphogenic; therefore, a weak responsiveness to lighting conditions was observed.

It is well known that one of the important factors necessary for the growth, development and productivity of plants is the intensity and spectral composition of light. In conditions of insufficient supply of sunlight, the process of photosynthesis is disrupted, the growth, development, productivity, and resistance of plants are reduced [50,51,52].

In the literature, there are numerous works devoted to the study of the intensity and spectral composition of light for growth and development, photosynthesis and plant productivity [38,39,40,41,42,43,44,45]. Light also acts as an effective regulator that controls plant morphogenesis during their development in vitro [38,39,40]. Various authors have found that cultivation of cell cultures under conditions with the use of red light leads to the stimulation of growth, both of the aerial part and of the roots, in comparison with cultivation in white and blue light [53,54]. In addition, it has been shown that red light enhances the synthesis of carbohydrates in the leaves, while blue light enhances the synthesis of proteins [40,53]. Our studies have shown that the use of different lighting conditions (FR > R, FR = R, FR < R) have a significantly stimulating effect on the accumulation of inulin in callus. Moreover, the maximum value of inulin in callus (7.55–7.95%) was obtained in the treatment 7.5 mg/L IAA with FR > R. According to the data obtained, the best combinations of studied factors for callus growth were 7.5 mg/L NAA with FR > R and 7.5 mg/L IAA with FR < R.

## 5. Conclusions

The cultivation of callus cells is one of the uses of the PGRs. The nature and concentration of PGRs in culture media has an effect on morphogenesis and secondary metabolite biosynthesis. The studies performed allowed us to conclude that in order to obtain a well-proliferating, non-morphogenic callus, the presence of NAA in the nutrient medium is necessary, and IAA to obtain regenerated plants from callus. Such plants can be initial material for the selection of new forms of chicory.

The light treatment had a significant effect on the inulin content in callus. The FR > R treatment with PPFD = 142 (±10) μmol/m^2^s increases the content of inulin in callus cells. On the other hand, the ability of callus cells to accumulate inulin also depends on the type of auxin used. The increased content of inulin in callus obtained in the medium with IAA can be explained by the appearance of meristematic foci, in contrast to the medium with NAA, on which non-morphogenic callus was formed.

## Figures and Tables

**Table 1:** Effect of different concentrations of various auxins (IAA and NAA) in combination with 2.0 mg/L BA on the growth of callus at different subcultures.

**Auxin**	**Auxin Concentration, mg/L**				
	5.5	6.5	7.5	8.5	9.5
4th subculture					
IAA	0.77 ± 0.03 d	0.48 ± 0.03 b	0.22 ± 0.01 a	0.54 ± 0.03 bc	0.77 ± 0.03 d
NAA	0.78 ± 0.03 d	1.56 ± 0.41 ef	1.08 ± 0.38 de	1.32 ± 0.51 def	0.66 ± 0.03 c
5th subculture					
IAA	0.92 ± 0.04 cde	0.84 ± 0.04 cd	0.63 ± 0.03 a	0.69 ± 0.03 ab	0.91 ± 0.04 cde
NAA	0.60 ± 0.03 a	0.80 ± 0.04 c	1.24 ± 0.58 def	1.24 ± 0.55 def	0.90 ± 0.04 cde

Mean (callus fresh weight, g) ± standard error (SE); At each subculture, means followed by a different letter are significantly different at an alpha level of 0.05 according to the Fisher’s Least Significant Difference (LSD) test.

**Table 2:** Effect of different auxins (2.0 mg/L BA in combination with 7.5 mg/L NAA or 7.5 mg/L IAA) on the inulin content (%) in chicory callus.

Auxin	Extraction Medium	Mean Optical Density	Fructose-Containing Sugars, %	Inulin Content, %
		4th subculture		
NAA	Alcohol	0.0387	4.2928	1.15 a
	Water	0.0491	5.4464	
IAA	Alcohol	0.0435	4.8215	5.04 b
	Water	0.0889	9.8612	
		5th subculture		
NAA	Alcohol	0.0407	4.3788	1.23 a
	Water	0.0463	5.6002	
IAA	Alcohol	0.0465	4.8441	5.27 b
	Water	0.0859	9.6449	

Means ± SE; At each subculture, means followed by a different letter are significantly different at an alpha level of 0.05 according to the Fisher’s Least Significant Difference (LSD) test. Values of inulin content were arcsin *√*X transformed prior to statistical analysis.

## Data Availability

Not applicable.
